# Stress-Induced Lipocalin-2 Controls Dendritic Spine Formation and Neuronal Activity in the Amygdala

**DOI:** 10.1371/journal.pone.0061046

**Published:** 2013-04-09

**Authors:** Anna E. Skrzypiec, Rahul S. Shah, Emanuele Schiavon, Eva Baker, Nathan Skene, Robert Pawlak, Mariusz Mucha

**Affiliations:** 1 University of Exeter Medical School, Exeter, United Kingdom; 2 Department of Cell Physiology and Pharmacology, University of Leicester, Leicester, United Kingdom; 3 Department of Neurosurgery, Wessex Neurological Centre, Southampton, United Kingdom; 4 Wolfson Institute for Biomedical Research and Department of Cell and Developmental Biology, University College London, London, United Kingdom; 5 Wellcome Trust Sanger Institute, Cambridge, United Kingdom; Max Planck Institute of Psychiatry, Germany

## Abstract

Behavioural adaptation to psychological stress is dependent on neuronal plasticity and dysfunction at this cellular level may underlie the pathogenesis of affective disorders such as depression and post-traumatic stress disorder. Taking advantage of genome-wide microarray assay, we performed detailed studies of stress-affected transcripts in the amygdala – an area which forms part of the innate fear circuit in mammals. Having previously demonstrated the role of lipocalin-2 (Lcn-2) in promoting stress-induced changes in dendritic spine morphology/function and neuronal excitability in the mouse hippocampus, we show here that the Lcn-2 gene is one of the most highly upregulated transcripts detected by microarray analysis in the amygdala after acute restraint-induced psychological stress. This is associated with increased Lcn-2 protein synthesis, which is found on immunohistochemistry to be predominantly localised to neurons. Stress-naïve Lcn-2^−/−^ mice show a higher spine density in the basolateral amygdala and a 2-fold higher rate of neuronal firing rate compared to wild-type mice. Unlike their wild-type counterparts, Lcn-2^−/−^ mice did not show an increase in dendritic spine density in response to stress but did show a distinct pattern of spine morphology. Thus, amygdala-specific neuronal responses to Lcn-2 may represent a mechanism for behavioural adaptation to psychological stress.

## Introduction

Prolonged or traumatic stressful events influence neuronal morphology and function in distinct brain regions such as the amygdala and hippocampus, a process which may give rise to affective disorders. The amygdala consists of an almond-shaped group of nuclei located deeply within the medial temporal lobes and is responsible for fear memory formation, consolidation and retention [1]. During fear processing, the amygdala is undergoing dynamic changes including extracellular protease activation [2], proteolytic cleavage of synaptic receptors [3] and dendritic arborisation [4]. Such changes require the synchronization and tight control of gene expression and protein synthesis.

Stress hormone release can activate cascades of gene expression, affecting central nervous system function and animal behaviour. Upon agonist binding, glucocorticoid receptors translocate into the cell nucleus and bind to DNA glucocorticoid response elements to positively [5] or negatively [6] affect transcription of target genes. They may also alter neuronal plasticity by inducing synthesis or activation of extracellular proteases. Harada et al. [7] showed that corticosterone release in response to psychological stress increases the expression and release of neuropsin to regulate neuronal plasticity [8]. Stress hormones also influence gene expression indirectly through regulation of post-translational protein phosporylation by MAPK and Egr-1 pathways [9] including histone phosphorylation [10]. Other stress-induced histone modifications linked with the stress response such as acetylation [11] and methylation [12] can also change gene expression patterns.

Due to technical difficulties, most studies have focussed on regulation and expression of single or small group of genes responding to psychological stress. Here we took advantage of microarray technology allowing genome-wide analysis of gene expression in amygdala of acutely restrain-stressed mice. Among the positively regulated transcripts we found well known stress-related genes regulated by glucocorticoids including Sgk1 [13], Fkbp5 [3] and Map3k6 [14].

One of the genes highly upregulated in response to stress was lipocalin-2 (Lcn-2, 24p3 or NGAL), which is also known to be regulated by glucocorticoids [15]. Lipocalins are a family of over 20 small, secreted proteins serving different cellular functions [16]. They transport lipophilic molecules, form macromolecular complexes and act upon membrane receptors to modulate cell growth, metabolism and immune reactions [17], [18]. We have previously described the novel role of Lcn-2 in regulating neuronal morphology and excitability in the hippocampus [19]. We show here that Lcn-2 is highly upregulated by restraint stress in the mouse amygdala, where it also regulates dendritic spine density and neuronal excitability. Region-specific effects of Lcn-2 in the amygdala provide a novel mechanism for stress-induced neuroplasticity and may contribute to anxiety behaviour.

## Results

### Microarray Analysis of Restraint-stress Induced Genes in Mouse Amygdala

**Figure 1:**
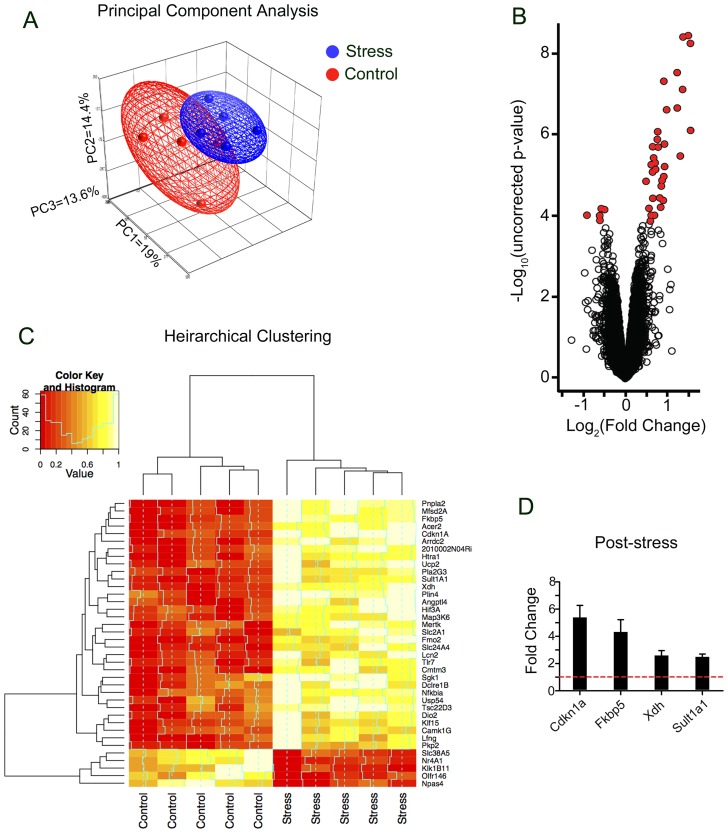
Microarray analysis of gene expression in the amygdala following acute restraint stress. **A**. Principal component analysis of microarray data obtained by hybridization of mRNA extracted from amygdalae of wild-type control (n = 5) and stressed (n = 5) mice. The figure represented the first three principal components of microarray analysis data (PC1, PC2 and PC3). The analysis of microarrays revealed the existence of two relative distinctive components of 5 metasets of control (red) and stress-affected (blue) gene expression patterns. **B.** The volcano plot depicting the range of gene expression fold-change (log_2_[fold change]) induced by restraint stress and corresponding p-value (-log10[uncorrected p-value]). Using the threshold cut-off of an absolute 1.3 fold-change and corrected p-value ≤0.05, statistically significant genes are marked in red. **C**. Hierarchical clustering of normalised signal intensities read from microarray probe sets. **D**. qRT-PCR verification of example genes identified as upregulated in response to restraint stress. The basal level of expression is marked by the red dashed line.

**Figure 2:**
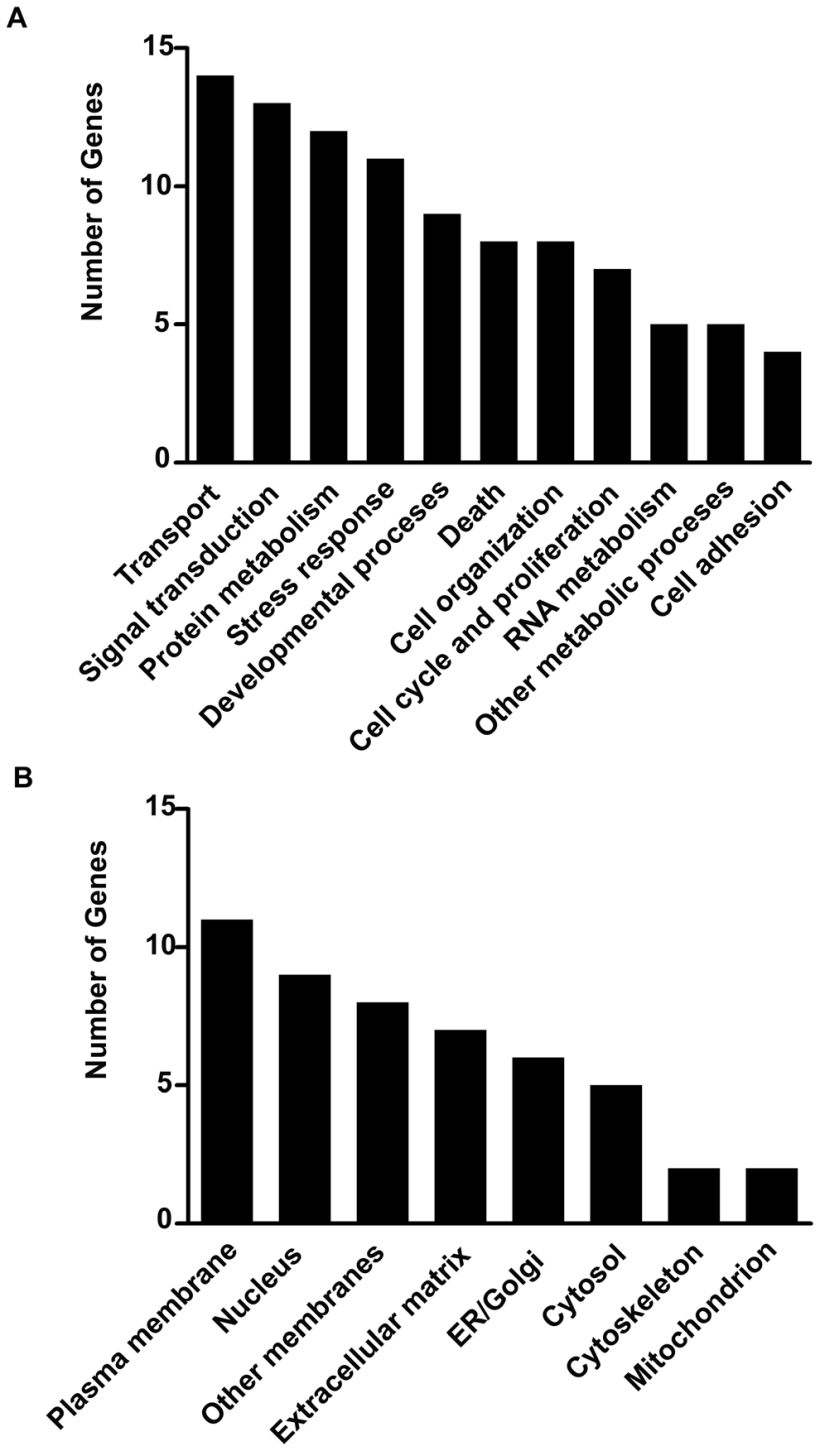
General characterisation of restraint stress affected transcripts. Stress affected transcripts divided according to their protein product function (**A**) and localisation (**B**).

**Figure 3:**
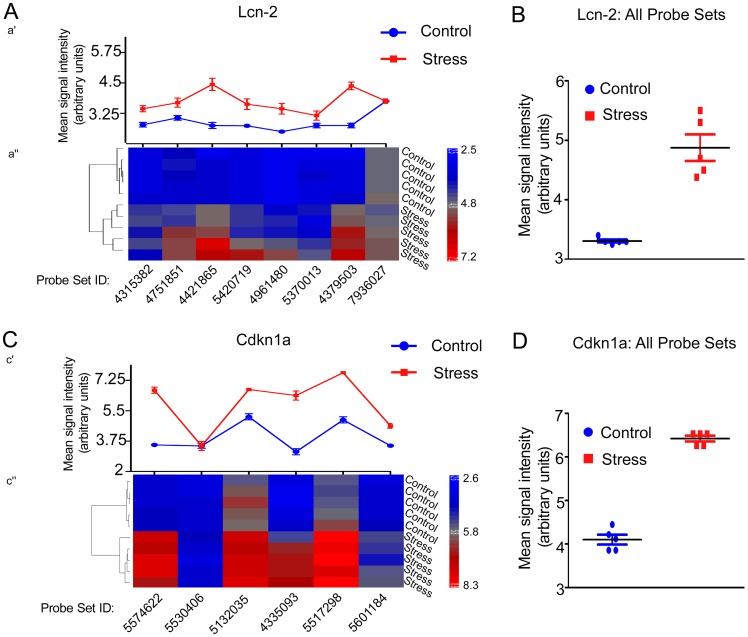
Alternative splicing analysis. Example of alternative splicing analysis of Lcn-2 (**A**,**B**) and Cdkn1a (**C**,**D**) genes expressed in response to restraint stress. Analysis of mean signal intensities read from probe sets annotated to 5′, 3′ untranslated regions (UTRs) and exons of Lcn-2 (a’ and a” respectively) and Cdkn1a transcripts (c’ and c’’ respectively) revealed no alternative splicing events in Lcn-2 transcript. Analysis of Cdkn1a transcript revealed reduced level of expression of probe set 5530406 annotated to untranslated exon 2 (Cdkn1a transcript variant 2, NM_001111099) in both control and stressed animals suggesting tissue specific alternative splicing unrelated to stress. Scatter plots of summarised intensities of all probe sets annotated to Lcn-2 (**B**) and Cdkn1a (**D**) transcripts revealed two relatively separate populations of stress related data points and no outliers significantly affecting splice variant analysis. Each point represents the mean of all probe set intensities annotated to the gene of interest from one array. Data presented as mean ±SEM.

To identify genes contributing to the stress response we subjected wild-type mice to acute restraint stress and performed genome-wide gene expression profiling in the amygdala using microarrays. The principal component analysis of obtained data revealed existence of two relatively distinctive subpopulations of data points resulting from restraint stress (Fig. 1A). Next, we verified the general distribution of stress-affected transcripts using volcano plot (Fig. 1B). We found that the restraint stress affected expression of specific gene (FC ≥1.3 and FC ≤ −1.3, adjusted p-value ≤0.05) (Fig. 1B–C and Table 1). The transcripts encoding nuclear and membrane proteins represented almost half of stress affected genes. This would be consistent with the membrane-to-nucleus signalling and activity-driven transcription contributing to stress-induced regulation of cellular responses (Fig. 2). Functional analysis revealed that the majority of the regulated genes were involved in the control of cellular transport, signal transduction, protein metabolism and the stress reponse. Several of these genes have been shown to be regulated by glucocorticoids (e.g. Fkbp5, Map3k6 and Sgk1) confirming the central role of these hormones in the adaptation of the central nervous system to stress (for review see [20]). Gene over- and underrepresentation analysis (Table 2) revealed the existence of 16 gene ontology functional/structural subcategories overrepresented significantly. We also examined microarray data looking for alternative splicing events induced by stress by comparing the mean signal of all probe sets annotated to all exons of stress regulated genes (Fig. 3). We found no stress-related alternative splice variants within the above group of genes.

**Table 1:** List of amygdalar transcripts affected by acute restraint-stress.

Gene Symbol	Entrez_gene_id	P-value	Adjusted P-value^BH^	Adjusted P-value^B^	Log (Fold-Change)
Fkbp5	14229	3.69E-09	2.35E-05	4.52E-05	1.49
Xdh	22436	4.01E-09	2.35E-05	4.90E-05	1.37
Cdkn1A	12575	5.76E-09	2.35E-05	7.05E-05	1.54
Mfsd2A	76574	3.01E-08	9.20E-05	3.68E-04	1.23
Pnpla2	66853	4.91E-08	1.20E-04	6.01E-04	0.91
Plin4	57435	7.83E-08	1.60E-04	0.001	1.359
Sult1A1	20887	2.24E-07	3.78E-04	0.003	1.232
2010002N04Rik	106878	2.47E-07	3.78E-04	0.003	0.980
Lcn2	16819	7.97E-07	0.001	0.010	1.547
Acer2	230379	8.55E-07	0.001	0.010	0.768
Fmo2	55990	1.33E-06	0.001	0.016	0.759
Angptl4	57875	1.72E-06	0.002	0.021	0.928
Arrdc2	70807	2.01E-06	0.002	0.025	0.648
Hif3A	53417	2.05E-06	0.002	0.025	0.779
Tlr7	170743	3.40E-06	0.003	0.042	1.303
Htra1	56213	3.77E-06	0.003	0.046	0.671
Cmtm3	68119	5.00E-06	0.004	0.061	0.706
Pla2G3	237625	5.56E-06	0.004	0.068	0.615
Ucp2	22228	6.17E-06	0.004	0.076	0.932
Mertk	17289	7.22E-06	0.004	0.088	0.717
Map3K6	53608	8.41E-06	0.005	0.103	0.647
Slc2A1	20525	1.08E-05	0.006	0.133	0.912
Tsc22D3	14605	1.36E-05	0.007	0.166	0.871
Slc24A4	238384	1.42E-05	0.007	0.174	0.491
Nfkbia	18035	1.86E-05	0.009	0.228	0.853
Pkp2	67451	3.64E-05	0.017	0.445	0.816
Klf15	66277	3.72E-05	0.017	0.455	0.652
Sgk1	20393	4.19E-05	0.018	0.513	0.900
Dio2	13371	6.20E-05	0.026	0.758	0.839
Lfng	16848	6.59E-05	0.026	0.806	0.561
Slc38A5	209837	6.69E-05	0.026	0.818	−0.570
Nr4A1	15370	7.07E-05	0.027	0.865	−0.492
Klk1B11	16613	9.70E-05	0.034	1.000	−0.911
Dclre1B	140917	9.75E-05	0.034	1.000	0.690
Usp54	78787	9.76E-05	0.034	1.000	0.614
Npas4	225872	9.94E-05	0.034	1.000	−0.608
Olfr146	258742	1.30E-04	0.043	1.000	−0.602
Camk1G	215303	1.35E-04	0.043	1.000	0.594

The list of transcripts affected by restraint stress as compared to control (stress-naïve) mice. Genes are organised by statistical significance (lowest to highest p-value). P-values were adjusted according to the Benjamini & Hochberg (^BH^) or Bonferroni (^B^) method. n = 5 microarrays per group, each microarray chip hybridised with pulled RNA extracted from 3 different animals.

**Table 2:** Statistical analysis of over-represented Gene Ontology groups of restraint-stress regulated transcripts.

Gene Ontology subcategory	Expected gene number	Observed gene number	Adjusted p-value
Cellular process	9.8499	22	0.0001
Metabolic process	6.2689	18	0.0001
Regulation of biological quality	1.2781	9	0.0002
Positive regulation of cellular process	1.4402	9	0.0005
Positive regulation of metabolic process	0.7786	7	0.0006
Cytoplasmic part	3.6616	13	0.0011
Positive regulation of biological process	1.618	9	0.0012
Cellular metabolic process	5.2917	15	0.0024
Response to chemical stimulus	0.972	7	0.0027
Protein binding	4.6657	14	0.0029
Positive regulation of macromolecule metabolic process	0.7006	6	0.0041
Positive regulation of cellular metabolic process	0.7214	6	0.0049
Catalytic activity	4.2409	13	0.0054
Cytosol	0.4925	5	0.0086
Response to stress	1.1836	7	0.0093
Cytoplasm	5.9065	15	0.0094
Cell part	12.533	22	0.0102
Cell	12.534	22	0.0103
Intracellular part	8.4557	18	0.0104
Intracellular	8.6594	18	0.0147
Response to stimulus	2.3055	9	0.0196
Regulation of apoptosis	0.6096	5	0.0232
Regulation of programmed cell death	0.6174	5	0.0246
Regulation of cell death	0.6286	5	0.0267

Sixteen Gene Ontology (GO) subcategories were over-represented in the group of 38 stress-affected genes. Only GO categories with 5 or more stress affected genes are shown. The p-values were adjusted according to the Bonferroni’s method. The subcategories were ordered accordingly to p-values.

### Lipocalin-2 is Upregulated by Stress and Expressed by Neurons in the Amygdala Complex

**Figure 4:**
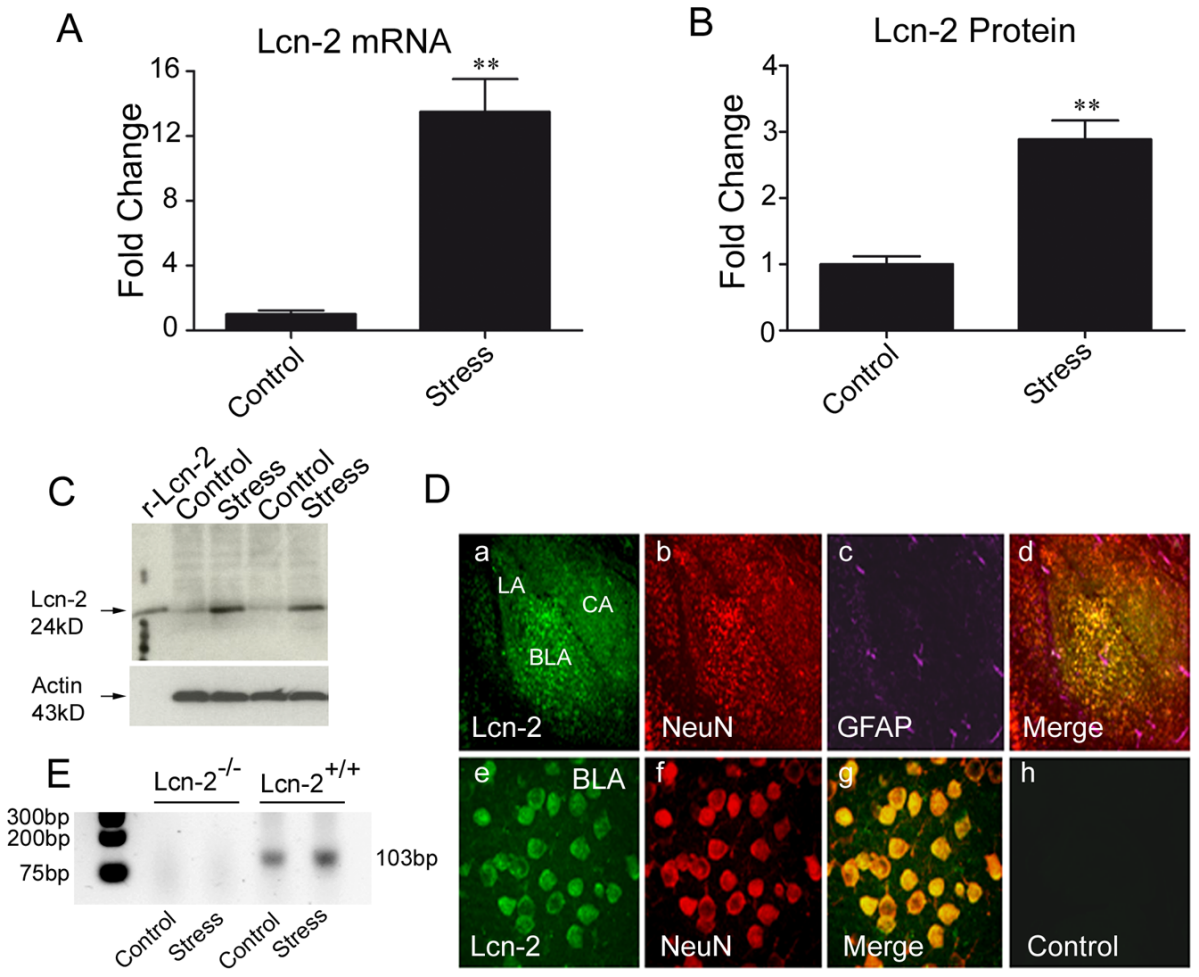
Lipocalin-2 is upregulated by psychological stress in mouse amygdala. Psychological stress induces lipocalin-2 gene expression N = 5, ** p<0.01 (**A**) followed by protein synthesis (**B** and **C**); R-Lcn-2– recombinant lipocalin-2 N = 3, ** p<0.01. Data are expressed as mean ± SEM. Panel C consist representative Western blot. Triple immunohistochemistry revealed (**D**) that Lcn-2 (green) is localised mostly within and nearby of neurons (a and e) co-localised with neuronal marker (b and f) NeuN (red) and to lesser extend with astrocyte marker (purple) GFAP (c and g) in the nucleus of basolateral amygdala. The secondary antibody showed no signal resulting from nonspecific binding (h). LA, Lateral Amygdala; BLA, Basolateral Amygdala; CA, Central Amygdala. Quantitative RT-PCR reaction confirmed lack of expression of Lcn-2 gene in Lcn-2^−/−^ animals (**E**).

Microarray analysis revealed lipocalin-2 (Lcn-2) as a highly upregulated gene in the amygdala in response to restraint stress (Table 1). Because of the technical complexity of the microarray design the results might be biased by either high homology among genes or cross-hybridization between probes, leading to either false-positive findings or underestimation of differentially expressed transcripts. To confirm our microarray data we performed qRT-PCR and found a ∼13-fold upregulation of the Lcn-2 gene in response to restraint stress in the amygdala (Fig. 4A; n = 5; p<0.001). Similarly, Western blotting demonstrated an upregulation of Lcn-2 protein after stress (Fig. 4B and 4C; n = 3; p<0.01) suggesting that the transcriptional regulation of the Lcn-2 gene is accompanied by analogous changes in protein synthesis.

Next, we performed immunohistochemistry to determine where Lcn-2 is primarily expressed in the amygdala. We found high levels of Lcn-2 in the central nucleus and the basolateral complex of the amygdala (Fig. 4D a–d). We also detected considerable amounts of Lcn-2 in the brain parenchyma (not shown) consistent with previous reports of its extracellular release [21]. We performed the immunohistochemistry on both stressed and stress-naïve animals, however we saw no obvious differences in signal localisation (not shown).

Neuronal adaptation to external stimuli can be modulated by a variety of cell types including vascular components or glial cells [22]. To investigate in what cell types Lcn-2 was expressed we performed triple staining using cell-type specific markers (Fig. 4D e–h). Lipocalin-2 co-localized mainly with a neuronal marker NeuN and to a much lesser extent with the astrocyte marker glial acidic fibrillary protein (GFAP) indicating that it was predominantly expressed by neurons.

### Lipocalin-2 Regulates Stress-induced Changes in Spine Density and Function and Regulates Neuronal Excitability

**Figure 5:**
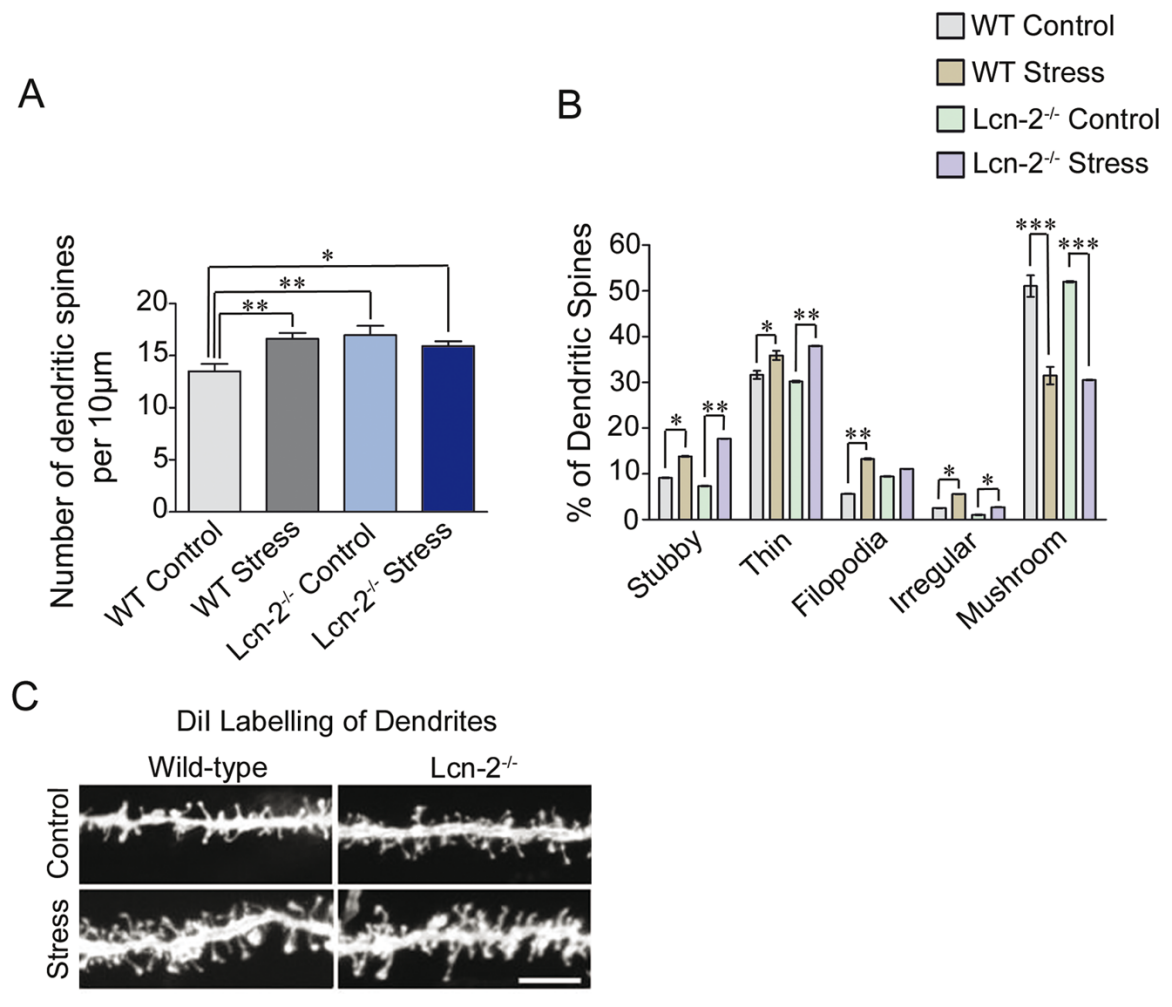
Lipocalin-2 regulates basal and stress-induced changes in dendritic spine density. Dendritic spine density in DiI-labeled neurons was analyzed in basolateral amygdala of wild-type and Lcn-2−/− mice before and after restraint stress. (**A**) Stress caused an increase in spine density in the neurons of BLA in wild-type mice reaching density observed in Lcn-2-deficient stress naïve mice. (**B**) Stress induced also significant decrease in proportion of mushroom spines observed in both wild-type and Lcn-2−/− strains. Those changes were accompanied by increase in other morphological groups of spines (B). Panel C represents the example of DiI stained neurons. *p<0.05; **p<0.01; ***p<0.001. Data are expressed as mean ± SEM.

Neuronal plasticity is, in part regulated by dendritic spine density and morphology. We examined whether Lcn-2 affects dendritic spine density *in vivo* in amygdala. Basolateral amygdala neurons were labelled with DiI in wild type and Lcn-2^−/−^ mice and spine density and morphology examined according to the previously published criteria [23]. Stress-naive Lcn-2^−/−^ animals showed higher spine density in the basolateral complex of the amygdala (p<0.01) when compared to wild-type controls (Fig. 5A). Baseline spine morphology did not differ between the genotypes (Fig. 5B). To investigate whether lipocalin-2 affects experience-driven spine plasticity we performed DiI labelling in Lcn-2^−/−^ and wild-type amygdala neurons *ex vivo* after stress (Fig. 5A). Because single session of restraint stress appeared not to be sufficient to trigger persistent changes at dendritic spine level in amygdala (data not shown), we decided to perform 3 restraint sessions to assess if such changes could be precipitated by repeated stress. We found no increase in spine density after stress in amygdala of Lcn-2^−/−^ animals while in wild-type mice we found a ∼15–20% increase in spine density, confirming previously reported observations [24]. In wild-type animals, after stress, the spine density reached the density of spines observed in stress-naïve Lcn-2^−/−^ mice (Fig. 5A F_(3,67)_ = 5,436).

We next considered a possibility that Lcn-2 might regulate either basal or stress-induced changes in spine morphology/maturation. We found that stress caused a decrease in the proportion of the mushroom spines in the basolateral complex of the amygdala, equally pronounced in both genotypes (Fig. 5B-C). There was also a concomitant increase in the proportion of stubby and thin spines in response to stress in both Lcn-2^−/−^ and wild type mice. Both groups also showed an increase in the proportion of irregular spines after stress, but a significant rise in filopodia was only seen in wild-type animals (Fig. 5B).

**Figure 6:**
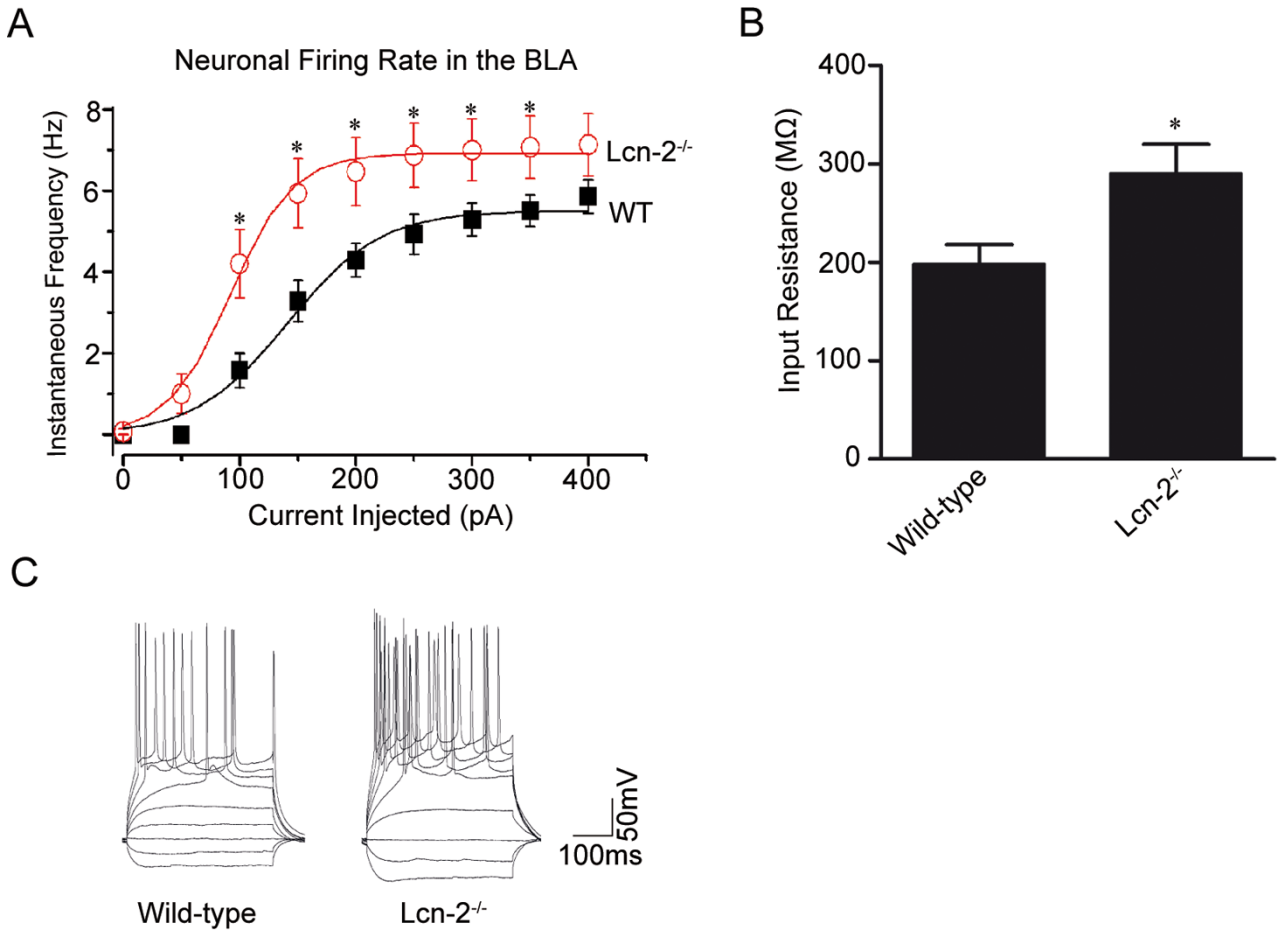
Region-specific control of neuronal excitability by lipocalin-2. Current-clamp experiments revealed that neuronal firing rate in the basolateral amygdala is higher in Lcn-2−/− mice when compared to Lcn-2+/+ animals. Voltage responses were recorded by current steps from −100 to +600 pA in 50 pA (starting membrane potential −80 mV) from principal neurons of the basal nucleus of the amygdala. Number of action potential spikes was counted as a function of depolarizing current injection (**A**). Disruption of the lipocalin-2 gene significantly increased action potential firing rate in Lcn-2−/− animals (p<0.01 at 150 pA; p<0.05 at 200 pA). Panel **B** shows a significant increase in the mean input resistance in Lcn-2−/− mice compared to wild-type animals. *p<0.05; Panel **C** is example of neuronal firing trace. Data are expressed as mean ± SEM.

Changes in dendritic spine density or morphology may strongly modify electrophysiological properties of neurons [25]. Thus, we examined whether lipocalin-2-induced changes in dendritic spine phenotype could alter neuronal excitability in current clamp recordings. We found that neurons from the basolateral amygdala of Lcn-2^−/−^ mice were more excitable and fired more action potentials than neurons from wild-type mice under the same conditions (Fig. 6A–C), consistent with a higher number of dendritic spines in Lcn-2 deficient animals.

## Discussion

Stressful events activate multiple metabolic pathways enforcing neuroplastic changes on synaptic and neuronal levels to enhance learning and memory [26], [27]. Nonetheless, prolonged or traumatic stress may lead to maladaptive forms of neuroplasticity and thus affect learning and memory [28], [29], [30].

The basolateral amygdala plays a role in processing and storing emotional aspects of experiences, allowing fast recognition and classification of experiences as positive or aversive, and facilitating an appropriate response to environmental stimuli. As such, detection and characterisation of specific neuromodulators affecting amygdala function may provide a target for drug design in conditions such as depression [31] or post-traumatic stress disorder [4]. To identify candidate neuromodulators we undertook genome-wide microarray assay and found that the transcript for lipocalin-2 showed the highest level of upregulation in response to restraint stress in the mouse amygdala.

Lipocalin-2 is a member of a small extracellular protein family, binding and transporting small hydrophobic molecules such as bilins, retinoids, lipids and steroids [32], [33], [34]. Lipocalins share similar protein architecture and to some degree amino acid sequence homology [16], [35]. They play important role in diverse processes including cell viability, cancer invasiveness, apoptosis and angiogenesis [36], [37], [38], [39].

In the central nervous system, it has been shown that lipocalin-2 is a modulator of deramification and apoptosis of activated microglial cells [40], reactive astrocytosis in response to brain injury [21] and increased plasma Lcn-2 levels have been shown to positively correlate with mild cognitive impairment [41]. Increased levels of lipocalin-2 considered together with its iron transport abilities may partially explain the accumulation of iron in brain associated with Alzheimer’s disease [42], Parkinson’s disease [43] and multiple sclerosis [44]. Devireddy and colleagues [45] showed that Lcn-2 binds to extracellular iron and is transported into cells where it releases iron for storage. However, low extracellular iron levels cause internalisation of free Lcn-2 (Apo-Lipocalin), which then binds intracellular iron and transports it to the extracellular space. In neurons, this decrease of internal iron concentration activates *Bim*, a proapoptotic member of the *Bcl-2* family of cell-death regulators, and induces apoptosis.

Psychological stress triggers changes in neuronal morphology in stress-processing brain areas like the basolateral amygdala and hippocampus. Vyas and colleagues [46] have shown that chronic unpredictable stress causes atrophy of dendritic processes in hippocampal CA3 pyramidal neurons accompanied by hypertrophy of dendritic processes in basolateral amygdala.

We have previously shown Lcn-2 gene to be upregulated in the hippocampus following stress, and that cultured hippocampal neurons treated with Lcn-2 purified protein had a reduced dendritic spine density [19]. We also found that lipocalin-2 affects spine density and morphology by changing the actin turnover within spines, an effect which was dependent on Lcn-2 iron binding ability.

Here we have shown that stress-naïve Lcn-2 knockouts have a higher spine density compared to WT mice in the amygdala (as also described in the hippocampus), and this is associated with an increase in neuronal excitability. However, there are several important differences in the phenotype of stress-induced changes in neuronal morphology between the hippocampus and amygdala in Lcn-2^−/−^ and wild-type mice.

In the amygdala, there is no significant increase in spine density after acute stress in Lcn-2^−/−^ mice, but stress does increase spine density in wild-type mice by 15–20% (to a level comparable to seen in pre-stress Lcn-2 knockouts). By comparison in the hippocampus, both Lcn-2^−/−^ and wild-type mice show an increase in spine density following acute stress. In the hippocampal CA3 region, this increase is similar in magnitude in both groups, while in CA1 neurons the increase is greater in Lcn-2^−/−^ mice compared to that seen in wild-types [19].

Acute stress also has a differential effect on the expressed dendritic spine phenotype. In the amygdala, stress causes a reduction of mushroom spines and a concomitant increase in stubby, thin and irregular spines in both Lcn-2 knockouts and wild-type mice, and increased filopdia in wild-type mice only. Post-stress hippocampi of lipocalin-2 knockouts show an increase in mushroom spines in CA1 and CA3 neurons, and a concomitant reduction in stubby and thin spines. In contrast, wild-type hippocampi showed an increase in stubby and thin spines in CA1 and CA3 regions without any change in mushroom spine frequency after stress.

The increase in the number of immature, neuroplastic spines (filopodia and thin) which are believed to have a potential role in memory formation [47] as well as a concomitant decrease in mushroom spines (believed to be responsible for memory storage) in wild-type amygdala after stress suggests their involvement in fear memory selection. Based on those findings we hypothesize that decrease in percentage of mushroom synapse-prone spines may be a part of adaptation process leading to decrease fear/stress associated memories while a plastic population of thin and filopodia spines kept in a “potential state” to form mushroom spines represents the reserve neural substrate for memory formation and storage. The absence of a rise in filopodia in Lcn-2 knockout mice may account for previous findings which show that they are more anxious in response to stress when compared to wild-type controls [19], suggesting enhanced emotionality and/or memory storage. In addition to our findings in the hippocampus, the evidence presented here suggests that the basolateral amygdala of lipocalin-2 deficient mice may also contribute to increased anxiety-like behaviour through aberrant dendritic spine plasticity and higher neuronal excitability.

Lipocalin-2 affects spine density, electrophysiological properties of neurons as well as stress-related animal behaviour and in future it will be crucial to clarify the metabolic pathways involved in this phenomenon. As such, performing microarray analysis in control and stressed lipocalin-2 knockouts and comparing to our own data could help identify co-regulated transcripts which are key in regulating responses to stress and anxiety.

In summary, distinct, region-specific neuronal changes produced by lipocalin-2 upon stress in the amygdala and hippocampus contribute to our understanding of adaptive and maladaptive processes that may underlie the development of stress-related psychiatric disorders.

## Materials and Methods

### Animals

#### Ethic statement

The experiments were approved by the UK Home Office and the UoL Ethical Committee.

Experiments were performed on three-month old male wild-type (C57/BL6) or Lcn-2^−/−^ mice (generous gift of Dr. Alan Aderem) [17] backcrossed to C57/BL6. Mice were genotyped as described [17]. Animals were housed three to five per cage in a colony room with a 12 hour light/dark cycle (lights on at 7AM) with *ad libitum* access to commercial chow and tap water.

### Restraint Stress

Prior to experiments mice were kept undisturbed for one week in their home cages. Restraint stress was performed during the light period of the circadian cycle as described [48]. Control animals were left undisturbed, and stressed animals were subjected to a six hour restraint stress session either once or on three consecutive days in a separate room. The mice were placed in their home cages in wire mesh restrainers secured at the head and tail ends with clips. A single session of restraint stress was applied before microarray studies, qRT-PCR analysis and western blotting whereas 3 consecutive restraint sessions were performed prior to spine density and morphology studies.

### Microarray Study

Amygdalae were dissected from wild-type mice, either naïve or subjected to a 6 hour restraint stress (15 animals per group) using a dissecting microscope in ice-cold ACSF buffer (25 mM glucose, 115 mM NaCl, 1.2 mM NaH_2_PO_4_·H_2_O, 3.3 mM KCl, 2 mM CaCl_2_, 1 mM MgSO_4_, 25.5 mM NaHCO_3_, pH 7.4). Tissue samples were kept submerged in RNAlater (Invitrogen) at −20°C before later processing Total RNA was extracted (RNeasy Lipid Tissue Mini Kit, Qiagen) and the ribosomal fraction reduced (RiboMinus Kit, Invitrogen). RNA integrity has been verified by electrophoresis using Agilent Bioanalyser 2100 (Agilent Technologies). RNA pulled from 3 animals has been reverse-transcribed and hybridized with GeneChip Mouse Exon 1.0 ST Array (Affymetrix). The quality of the Mouse Exon 1.0 ST Array data was checked according to the Affymetrix guidelines using the Expression Console software (Affymetrix). The microarray analysis method was performed using a combination of Affymetrix Powertools, Partek Genomic Suite (Partek, St. Louis, MO, USA), R platform, Bioconductor, and Limma [49] software. The general schema of analysis followed is as previously described [50]. The CEL files were read and processed with apt-probeset-summarize, and normalized using Robust Multichip Average-Sketch. Only the probes from the core annotation set with cross hybridization values of 1 were used. If at least half of the probes for a gene were not detected above background (p0.05) in at least three arrays from one class the gene was dropped from further analysis (reducing the total number of genes studied from 23,332 to 12,230 which is in line with the number of genes expected to be expressed in the brain)">50. The CEL files were read and processed with apt-probeset-summarize, and normalized using Robust Multichip Average-Sketch. Only the probes from the core annotation set with cross hybridization values of 1 were used. If at least half of the probes for a gene were not detected above background (p0.05) in at least three arrays from one class the gene was dropped from further analysis (reducing the total number of genes studied from 23,332 to 12,230 which is in line with the number of genes expected to be expressed in the brain). Partek Genomic Suite was used for primary component analysis and alternative splicing analysis, Apt-Midas was used for verification of differential splicing events, and Limma was used to look for differential gene expression with Benjamini and Hochberg or Bonferroni corrections applied to all p-values. CEL files has been deposited at MAGE-TAB ArrayExpress (http://www.ebi.ac.uk/cgi-bin/microarray/magetab.cgi, accession number E-MTAB-1519).

Gene function and gene product localisation analysis was performed using MGI Gene Ontology GO Slim Chart Tool (http://www.informatics.jax.org/gotools/MGI_GO_Slim_Chart.html) with default parameters. The gene under- and overrepresentation analysis was performed using Gene Trail software [51] (http://genetrail.bioinf.uni-sb.de/index.php) with the minimal number of genes per group set as 5 and Bonferroni correction of p-values.

### qRT-PCR

Control and stressed mice were anaesthetised intraperitoneally using sodium pentobarbital (50 mg/kg) and perfused transcardially with ice cold PBS. The brains were removed and dissected in ice-cold PBS using a vibrating microtome (Campden Instruments). The amygdalae were dissected from a coronal slice −0.58 to −2.3 mm relative to Bregma. Samples were submerged in RNAlater (Invitrogen) at −20°C, homogenised in QIAzol lysis reagent (QIAgen) and total RNA was isolated (RNeasy Lipid tissue mini kit, QIAgen). A total of 2 µg of RNA from each sample was converted to cDNA using Superscript III (Invitrogen) and oligo (dT) primers according to manufacturer’s instructions. To verify the microarray data RNA samples from 5 control and 5 stressed animals (i.e. 5 out of 15 for each group) used previously for array hybridisation were randomly chosen, reverse-transcribed and used as templates for qRT-PCR.

qRT-PCR for Lcn-2 was performed using Chromo4/PTC-200 thermal cycler (MJ research) as follows: 95°C for 15 minutes, 94°C for 15 seconds, 55°C for 30 seconds, steps 2–4 were repeated 40 times, 72°C for 30 seconds. The following primers were used for Lcn-2 gene expression quantification: 5′-CCCCATCTCTGCTCACTGTC and 5′-TTTTTCTGGACCGCATTG. The Cdkn1a, Fkbp5, Xdh and Sult1a1 were quantified using primers: 5′-AAAGTGTGCCGTTGTCTCTTC and 5′-CAAAGTTCCACCGTTCTCG, 5′-CCCTGGTGAAGATGCAGAG and 5′-GGGGTCAATGCCAAACTTAG, 5′-CTCCAAGTATGACCGCCTTC and 5′-TCCTATGCCTTCCACAGTTG, 5′-GCTCAGAACCCCAGCAAC and 5′-CTCCATTGTCTCCGCAAAG respectively. No-template control reactions were run in parallel.

### Western Blotting

Control and stressed mice (different than in microarray study) were anaesthetised intraperitoneally using sodium pentobarbital and perfused transcardially with ice cold PBS. The brains were removed and the amygdalae dissected as above, homogenized in 0.1 M Tris, 0.1% Triton X-100, pH 7.4, containing protease inhibitors (Complete, Roche) and the protein concentration was adjusted to 2 mg/ml using the Bradford method (Pierce). Reduced (DTT) and denatured (100°C for 5 minutes) samples (40 µg per lane) were subjected to SDS-PAGE electrophoresis and transferred onto nitrocellulose membrane. After blocking (5% skim milk for 1 h at RT) and washing with TBS-T (3×5 mins) the membranes were probed with goat anti-Lcn-2 antibody (R&D, 1∶500) overnight at 4°C. The membranes were then washed in PBS-T (3×10 min) before incubation with a relevant HRP-conjugated secondary antibody (Vector Labs, 1∶1000, 1 hr, RT). The signal was developed, after washing with TBS-T (6×10 min), using a Western Blot Luminol Reagent (Santa Cruz). To normalise the results the membranes were stripped, blocked, washed as above and re-blotted using mouse anti-β-actin antibody (Sigma, 1∶2500, 1 hr, RT).

### Immunohistochemistry

Mice were anaesthetised with sodium pentobarbital and transcardially perfused with ice-cold phosphate buffered saline (PBS) containing protease inhibitors (Complete, Roche) followed by ice-cold 4% paraformaldehyde (Sigma) in PBS. The brains were dissected and fixed in 4% paraformaldehyde in PBS overnight at 4°C. The paraformaldehyde was washed out and 70 µm thick coronal sections were collected using vibrating blade microtome. Brain sections were preincubated in PBS-T (PBS solution containing 0.5% bovine serum albumin, 0.02% Triton X-100 and normal goat serum (1∶500) for 5 hours at room temperature. Sections were then incubated with goat anti-Lcn-2 antibody (R&D, 1∶500) along with the mouse anti-NeuN (1∶200, Chemicon) and chicken anti-GFAP (1∶1000, Dako) overnight at 4°C in PBST. Next, the sections were washed for 8–10 hours with PBS-T and incubated overnight with appropriate AlexaFluor secondary antibodies (1∶500, Molecular Probes) in the same buffer. Control sections were processed as above but the primary antibodies were omitted. Sections were then washed in PBS-T for 5 hours, mounted on glass slides using Vectamount medium (Vector Laboratories), and photographed using Zeiss LSM 5 Exciter confocal microscope.

### Electrophysiology

Whole-cell recordings in current clamp configuration: coronal slices (250 µm) were obtained from 10–20 days old mice. The animals were anaesthetized with ketamine/xylazine (2∶1 ratio; 2.4 µl/g i.p.). Neurons were visualized by infrared videomicroscopy (CCD camera KP-M2RP) and recordings made from somata of principal neurons of the basal nucleus of the amygdala (n = 14 and n = 15 respectively). Principal neurons and interneurons were distinguished by their morphological and electrophysiological properties. Only neurons with a resting membrane potential below −50 mV were used. The cell membrane was clamped at −80 mV. Input resistance and instantaneous frequency were derived from traces in which cells were injected with 200 ms current pulses (−100 to +600 pA: 50 pA increments). The recording electrodes were borosilicate glass pipettes (2–4 MΩ) filled with the following solution (in mM): K-gluconate (130), KCl (4), EGTA (0.5), HEPES (10), glucose (5). ACSF composition (in mM): NaCl (124); KCl (5); NaH_2_PO_4_ (1.25); D-glucose (10);NaHCO_3_ (26); CaCl_2_ (2); MgSO_4_ (1). All the experiments were performed at 25°C. The recordings were amplified (multiclamp 700b Axon instruments), filtered (10 kHz), digitized 50 kHz (digidata 1440A Axon instruments) and stored in a PC. pClamp 10 (Axon Instruments) and Origin 7 (Microcal Inc.) software were used during data acquisition and analysis.

### DiI Labelling

DiI labelling was performed as previously described [52]. Briefly, the animals were anaesthetised and transcardially perfused with PBS followed by 1.5% paraformaldehyde. The brains were dissected and kept for 1 hour in 1.5% paraformaldehyde at RT and 170 µm coronal slices containing the amygdalae were cut on vibrating microtome. DiI cristals (Molecular Probes) were applied using borosilicate glass pipette and the slices were kept for 24 hours at RT. Sections were then fixed with 4% paraformaldehyde for 35 min, mounted on slides and dendritic tree visualized using Zeiss LSM5 Exciter confocal microscope. Spine densities were estimated along 650–1015 µm of secondary/tertiary dendritic branches per group. 550–800 spines per group were analyzed to assess spine morphology: mushroom spines: <2 µm in length, >0.5 µm in width and are connected to the dendritic shaft by a narrower portion (neck); stubby spines: <2 µm in length, >0.5 µm in width and lack a defined neck; thin spines: <2 µm in length, <0.5 µm in width and have a neck; filopodia: >2 µm in length, <0.5 µm in width and do not have a distinct spine head; irregular spines have more than one necks and/or heads. The researches analysing the spine density and morphology were unaware of tissue genotype and stress status.

### Statistics

Student t-test or analysis of variance (ANOVA) followed by Tukey’s post-test were used as appropriate. P values of less than 0.05 were considered significant.
